# Cysteine protease activation and apoptosis in Murine norovirus infection

**DOI:** 10.1186/1743-422X-6-139

**Published:** 2009-09-10

**Authors:** Linnzi M Furman, Walid S Maaty, Lena K Petersen, Khalil Ettayebi, Michele E Hardy, Brian Bothner

**Affiliations:** 1 Chemistry and Biochemistry, Montana State University, Bozeman, Montana, 59715 USA; 2 Veterinary Molecular Biology, Montana State University, Bozeman, Montana, 59715 USA

## Abstract

**Background:**

Noroviruses are the leading cause of viral gastroenteritis. Because a suitable *in vitro *culture system for the human virus has yet to be developed, many basic details of the infection process are unknown. Murine norovirus (MNV) serves as a model system for the study of norovirus infection. Recently it was shown that infection of RAW 264.7 cells involved a novel apoptotic pathway involving survivin.

**Results:**

Using a different set of approaches, the up-regulation of caspases, DNA condensation/fragmentation, and membrane blebbing, all of which are markers of apoptosis, were confirmed. Live cell imaging and activity-based protein profiling showed that activation of caspase-like proteases occurred within two hours of infection, followed by morphological changes to the cells. MNV infection in the presence of caspase inhibitors proceeded via a distinct pathway of rapid cellular necrosis and reduced viral production. Affinity purification of activity-based protein profiling targets and identification by peptide mass fingerprinting showed that the cysteine protease cathepsin B was activated early in infection, establishing this protein as an upstream activator of the intrinsic apoptotic pathway.

**Conclusion:**

This work adds cathepsin B to the noncanonical programmed cell death induced by MNV, and provides data suggesting that the virus may induce apoptosis to expand the window of time for viral replication. This work also highlights the significant power of activity-based protein profiling in the study of viral pathogenesis.

## Background

Noroviruses are the leading cause of non-bacterial gastroenteritis and cause roughly 23 million cases of foodborne illness annually in the United States alone (CDC, 1999). The virus infects people of all ages and is highly contagious amongst those susceptible to infection. The illness is best known for its fast-spreading outbreaks on cruise ships, college campuses, military bases, nursing homes, restaurants, and other semi-closed communities. While the majority of those infected with the virus recover in one to three days with no long-term sequelae, roughly 50,000 cases result in hospitalization annually in the U.S. with ~1% of those becoming fatal.

Noroviruses are a group of forty genetically heterogeneous viruses that belong to the *Caliciviridae *family. They are small RNA viruses with positive-sense, single-stranded genomes of ~7.7 kb. The particles are non-enveloped with T = 3 icosahedral symmetry, and are ~30 nm in diameter [1]. Noroviruses are the only group of animal viruses known to date whose capsid consists of a single protein form [2]. Attempts to grow human norovirus in cell culture have been largely unsuccessful [3], leaving many details of the replication and life-cycle unclear. Recently a murine norovirus strain (MNV-1) was identified [4] and has now become a model to study norovirus biology. MNV-1 has a tropism for dendritic cells and macrophages and grows to high titers in primary cells and in the cultured macrophage cell line RAW264.7 [5]. Based on the murine system, advances in elucidating cellular response to norovirus infection are being reported [6].

During infection, viruses commandeer cellular components such as trafficking proteins, membranes, enzymes, and organelles. Cells attempt to prevent this using a set of innate systems to combat viruses including establishing an antiviral state through interferon α/β signaling, RNAi, and apoptosis. Most viruses encode innate immune evasion strategies or even use cellular defense mechanisms to their own advantage. Programmed cell death (PCD) or apoptosis is one of the common pathways activated upon viral infection. Apoptosis is defined by a set of molecular characteristics including: chromatin condensation resulting from DNA fragmentation [7], cell shrinkage [8], membrane blebbing [9], phosphatidylserine exposure [10], and caspase activation [11]. While the signature markers of apoptosis are well characterized, intermediate forms of PCD have been described, but are not as fully understood. As a group, they lack one or more of the characteristics listed above [12]. Necrosis, or basic cell death, occurs without an orchestrated pattern when the cellular state is perturbed beyond rebound or physical damage occurs. Necrosis usually results in an inflammatory immune response due to leakage of cytoplasmic material.

The carefully controlled genetic and biochemical process of apoptosis is part of the cellular arsenal for fighting viral infection. PCD limits the function of cellular machinery involved in basic metabolic pathways and the time available for viral replication. Many viruses have evolved anti-apoptotic strategies to circumvent this mechanism to limit replication [13-16]. However, some viruses actually take advantage of PCD and the mechanisms involved. For example, PCD can provide an avenue for intercellular transfer of virus in membrane bound bodies, allowing undetected spread to neighboring cells. Over a dozen viruses have been documented to induce apoptosis during infection, each with its own mechanism for activation, and there are even more viruses that are known to inhibit apoptosis [12]. The mechanisms for inhibiting apoptosis target a handful of proteins, including caspases, Bcl-2, TNF (tumor necrosis factor), NFκB, PKR (dsRNA-dependent protein kinase), p53, and the oxidative stress pathway. Both NFκB and PKR stimulate interferons (IFNs), which are critical to the host's defense against viral infection. Most of the mechanisms that inhibit apoptosis through Bcl-2, TNF, p53 and NFκB ultimately lead to the prevention of caspase activation; these proteins indirectly activate the initiator caspases 8 and 9, and later, caspase-3. Activation leads to DNA fragmentation and apoptosis, where as blocking the initiator caspases can prevent apoptosis.

Interactions between virus and PCD signaling pathways are currently areas of high interest [14,17,18]. Viral-induced apoptosis has been demonstrated for a number of viruses including HIV [19], adenovirus [20], hepatitis C virus [21], herpes simplex virus [22], human papillomavirus [23], and influenza virus [24]. Apoptosis, as a feature of calicivirus infection, has recently been characterized by the increase in caspase-3 activity [10,25-27]. It has also been proposed that caspase-3 cleaves the norovirus polyprotein, suggesting that apoptosis might be required for viral replication [28]. More recently it was demonstrated that MNV infection induced apoptosis in cultured macrophages [29].

This study investigated cellular changes that occur in MNV infected cells by following the infection of murine macrophage cells (RAW 264.7). Analysis of infected cells confirmed the up-regulation of caspases, DNA condensation/fragmentation and membrane blebbing. Activity-based protein profiling (ABPP) showed that activation of cysteine proteases occurred within two hours of infection, rapidly followed by morphological changes to the cells. The addition of caspase inhibitors during infection led to rapid cellular necrosis and reduced viral titer. This study also found cathepsin B to be activated early in infection. Recent studies by Bok et al [29] found the MNV-cell interaction to be novel with respect to PCD in that the protein survivin is involved. Our results are consistent with their conclusions that the intrinsic apoptotic pathway becomes activated in RAW264.7 cells following infection by MNV and that cathepsin B acts upstream of cytochrome C release from mitochondria in this process. This is the first report of cathepsin B involvement in norovirus or viral infection in general.

## Results

### Caspase activation in MNV infected RAW264.7 cells

Recently it was shown that MNV-1 infection of RAW264.7 cells induced apoptotic changes. To establish the initial events during infection that lead to survivin down-regulation and PCD, we investigated caspase activation early in the infection process. Cell permeable activity-based probes linked to a fluorescent dye molecule were selected so that caspase activation could be assessed using live-cell imaging. We reasoned that this approach would be sufficiently sensitive to detect activation at early time points and would allow the progress of individual cells to be followed, thus avoiding population effects inherent in assays that require cell lysis.

**Figure 1:**
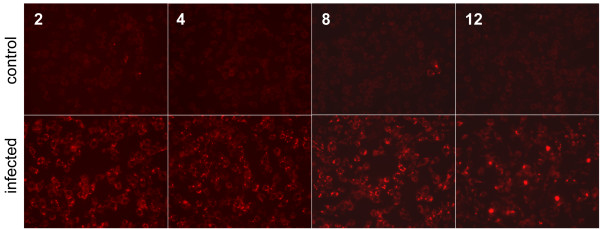
**Caspase activation during MNV-1 infection**. Mock-infected (top) and infected (bottom) RAW246.7 cells were incubated with a poly caspase specific activity-based probe 2, 4, 8, and 12 hours post infection (left to right). The poly-caspase probe is membrane permeable and linked to a sulforhodamine dye.

**Figure 2:**
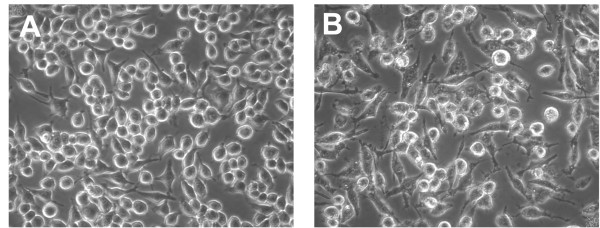
**RAW264.7 cell morphology changes during MNV-1 infection**. Brightfield cell images of mock-infected (A) and infected (B) cells 12 hpi. The cells in panel B have changed in overall shape and are vesiculated.

Total caspase activity was monitored using the FLICA probe SR-VAD-fmk, (a polycaspase probe reported to recognize all active capsases) at 2, 4, 8, and 12 hours post infection (hpi). Fluorescence microscopy showed a clear increase in caspase activity at 2 hpi compared with mock-infected control cells (Figure 1). Infected cells were consistently labeled between 2 to 10 hpi with nearly all cells showing signs of positive labeling. At 12 hpi, intensely stained cells were observed and a small number had lifted from the culture plate. A few positively labeled cells were visible on the control plates, but the number of labeled cells never exceeded more than a small percent of the total. The positive signal at 2 hpi was unexpected based on previous immunoblots that assessed caspase 3 and 9 cleavage [29] and canonical timing of apoptosis. A difference in these experiments was that here a caspase probe with broad specificity was used instead of the more selective probes that target specific members of the caspase family. MNV, like other RNA viruses, replicates on intracellular membranes and has been reported to cause a number of morphological changes to cells as membrane reorganization [5] and PCD progress [29]. Bright field images of cells 12 hpi in the FLICA experiment showed characteristic vesiculation and changes in shape (Figure 2) as previously described [5]. At this point in the infection cycle, infected cells were easily distinguished based on morphology from controls.

**Figure 3:**
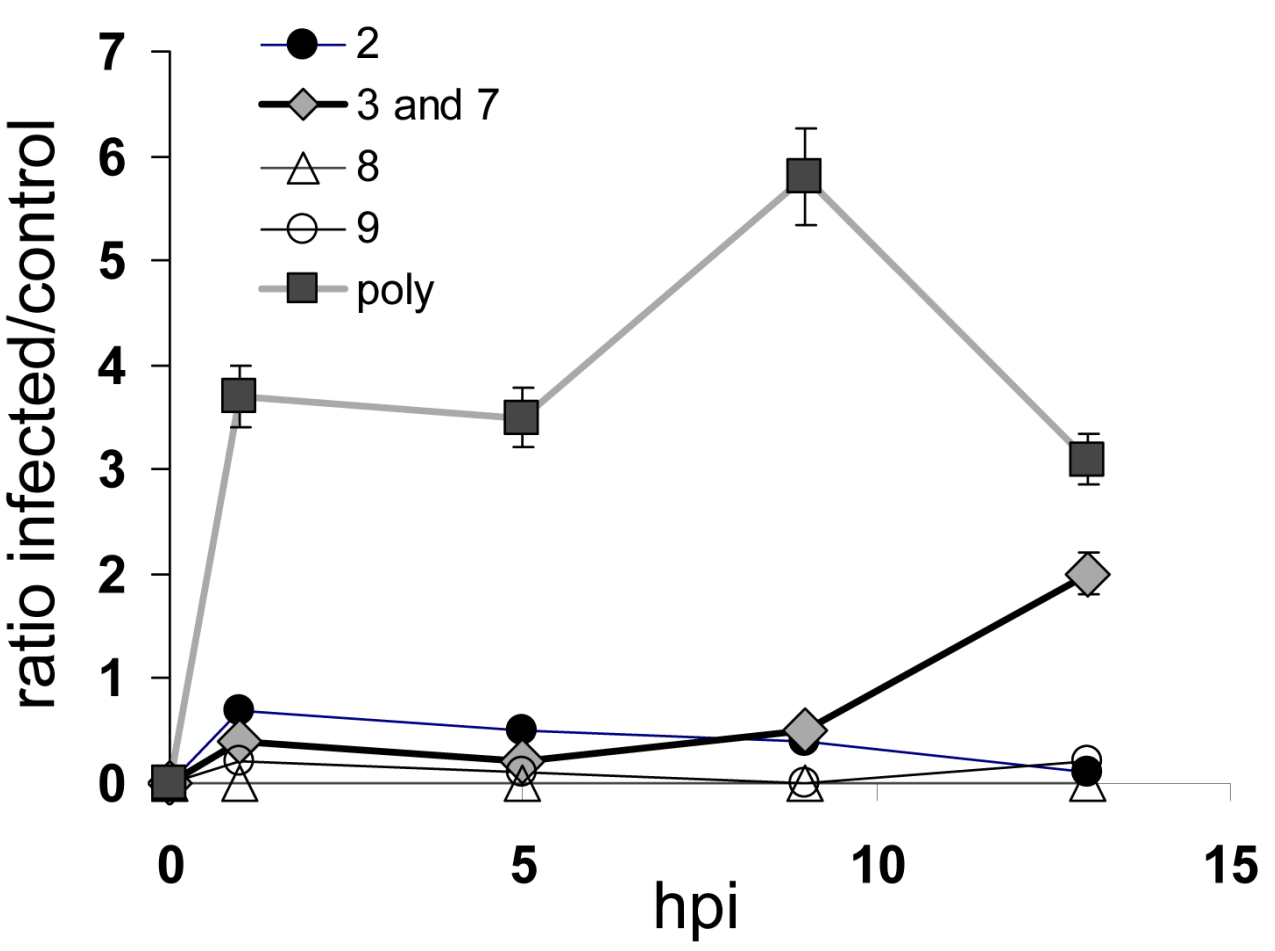
**Caspase activation after MNV-1 infection based on labeling of active proteases in live cells**. Individual classes of caspases were assayed for activity by labeling with membrane permeable covalent activity-based probes coupled to a fluorescent dye (n = 3). Activation is reported in relative fluorescence units.

Caspase activation follows a defined progression using intrinsic (mitochondrial) or extrinsic pathways involving initiator caspases 9 and 8, respectively. The two pathways merge at the effector capsase 3. To establish which caspase(s) were becoming activated, probes specific for caspases 2, 3/7, 8, and 9 were tested. In this experiment, cell lysates were analyzed using a fluorescent plate reader to obtain quantitative results. Infected cells reacted strongly with the broad spectrum activity probe after only 2 hours (Figure 3). By 13 hpi the signal from the poly probe was beginning to decrease while caspase-3/7 was being activated. The time course of caspase 3/7 activation is consistent with the data reported by Bok *et al *[29]. Of note, the previous study analyzed catalytic turnover of a substrate, whereas the experiments reported here measure the number of active enzymes present. Based on the activity curves (Figure 3), the signal from the poly caspase probe appears to be greater than the sum of the higher specificity probes prior to 13 hpi.

The induction of PCD in RAW264.7 cells by MNV is replication dependent, as inactivated virus failed to generate a response upon cell entry [29]. The mechanism behind this effect was not determined, however, failed caspase activation is a possible explanation. For example, caspase 3 is activated by initiator caspases 2, 8, and 9, and by blocking the initiator, caspase 3 is not activated. Survivin, an inhibitor of apoptosis (IAP) works by preventing activation of caspase 9. The caspase probes used in this study detect the number of active proteins in a cell, but are also caspase inhibitors. By forming a covalent bond with a catalytic residue side-chain the enzyme is irreversibly inhibited. Because of this, probes were added to the cells, allowed to react, and after washing were then visualized or harvested so that they are only reporting on cellular state at the time of addition.

### Caspase inhibitors accelerate cell death

**Figure 4:**
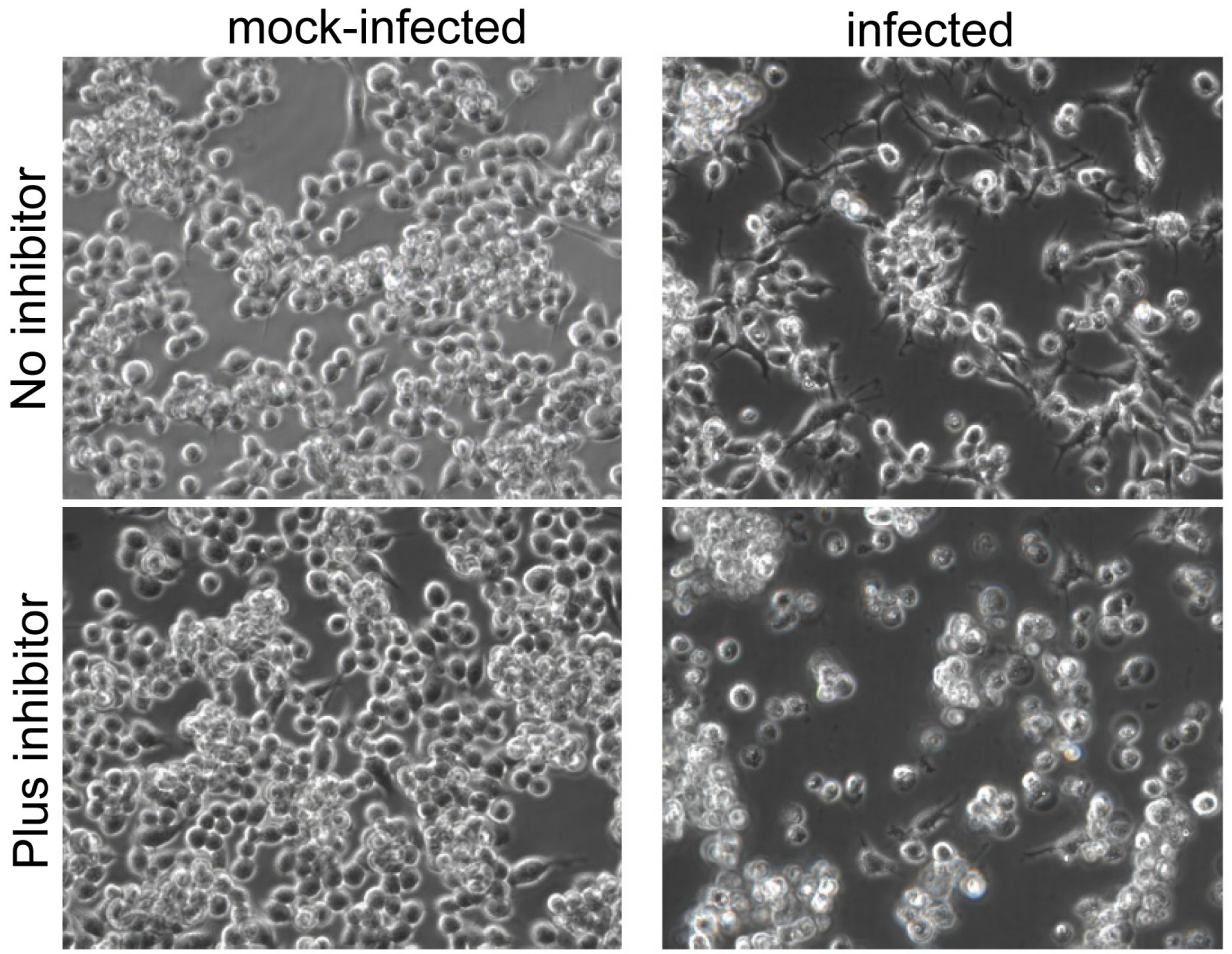
**MNV-1 infection with and without caspase inhibitors**. Cell morphology 18 hpi, contol (top row) and caspase-inhibited (bottom row) RAW 246.7 cells. Non-infected cells (left) were morphologically unaffected by the presence of the caspase inhibitor, Boc-D(MeO)-fmk. Cells infected in the presence of the caspase inhibitor (bottom right) were swollen and bulged, whereas those infected without the inhibitor shrank and broke apart.

**Figure 5:**
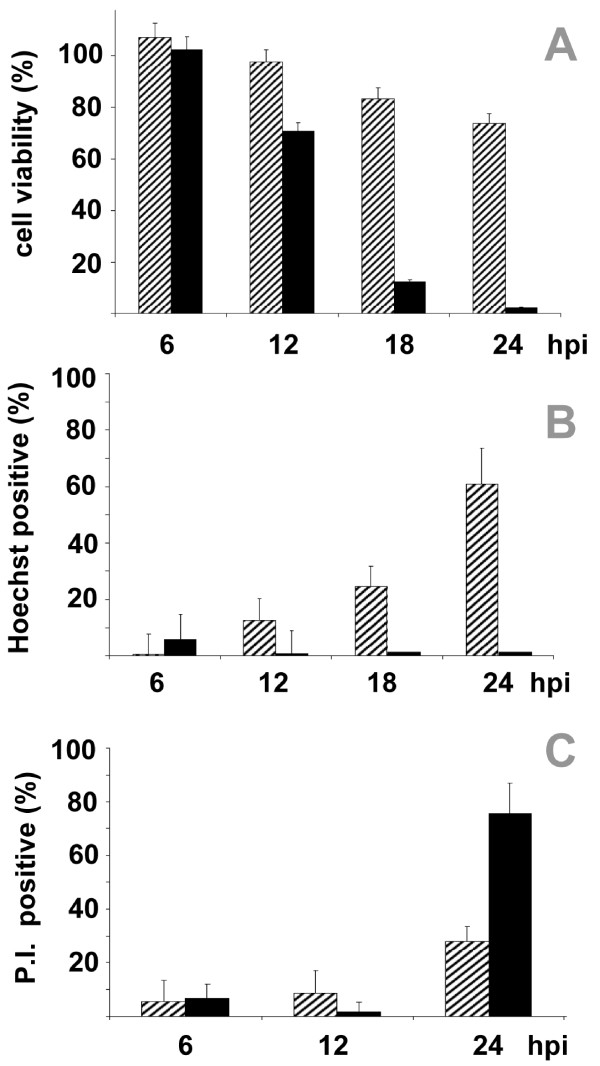
**Poly-caspase inhibitor alters the pathway of MNV-1 infection**. RAW 264.7 cells were infected with MNV-1 in the presence of the poly-caspase inhibitor, Boc-D-FMK (black bars), and without the inhibitor (striped bars). Trypan blue staining shows that cell viability decreases much more in the presence of inhibitor (A). Only cells infected in the absence of the caspase inhibitor show an increase in chromatin condensation as monitored with Hoechst stain (B). Cells infected in the presence of inhibitor lose membrane integrity more rapidly as monitored by propidium iodide (PI) staining (C). (n = 3 for all panels).

The rapid activation of caspases and changes in cell morphology described above led us to test whether MNV replication was dependent on PCD. RAW 264.7 cells were infected with MNV in the presence of the polycaspase probe, Boc-D-fmk. The cellular response to infection was very different in the presence of inhibitor and morphological differences were clearly visible 12 hpi. This was not due to the inhibitor alone, as uninfected cells treated with Boc-D-fmk remained viable and morphologically indistinct from control cells. Blebbing, swelling, and cytoplasmic leaking were evident in cells infected in the presence of the caspase inhibitor (Figure 4). In the absence of caspase inhibitors, smaller blebs and vesicles were visible at 12 hpi, after which the cells began to shrink and break apart into apoptotic-like bodies. The presence of activated macrophages, cells with elongated appendages, which were common during normal infection (Figure 4 top right panel and Figure 2B) were less frequent, possibly due to cells lifting from the plate earlier during the infectious cycle. Based on the microscopy, the presence of caspase inhibitors altered the infection process, leading to cell death that was better described as necrosis than apoptosis. Cells infected in the presence of the caspase inhibitor died more rapidly and fewer cells remained attached to the culture wells at 18 and 24 hpi. To confirm this, plasma membrane integrity was assessed over the course of infection with trypan blue staining. At 18 hpi less than 20% of the cells with inhibitor were viable, whereas greater than 80% where viable in the population that followed the normal infection process (Figure 5A). This confirmed that cells infected in the presence and absence of caspase inhibitor had dramatically different responses.

### Apoptosis vs necrosis

The steps and signs of PCD are usually straightforward to document. Necrosis, on the other hand, is broadly described by the lack of these features. To get a better understanding of the different processes that were occurring in the caspase inhibited and non-inhibited infected RAW264.7 cells, two additional stains were used. Hoechst staining is commonly used to detect chromatin condensation due to fragmentation during apoptosis because the nucleus stains an intense blue, whereas normal nuclear DNA shows a diffuse pattern of staining. PI can differentiate between live, dead, and apoptotic cells, as apoptotic cells will fluoresce an intense red, necrotic cell are a diffuse red, and healthy cells will remain unstained. By using these two stains in combination it is possible to differentiate between apoptotic and non-apoptotic cell death [30,31].

After infection, RAW 264.7 cells showed a steady increase in Hoechst staining over 24 hours, consistent with PCD (Figure 5B). Cells infected in the presence of the caspase inhibitor displayed no significant DNA condensation. These data indicate that DNA fragmentation, resulting in chromatin condensation, is minimal in cells infected in the presence of the caspase inhibitor, but extensive in cells infected without the inhibitor. Parallel cultures stained with PI showed signs of membrane permeability for both cell populations; however, cells infected in the presence of the inhibitor had a more drastic shift to membrane permeability than those infected without the inhibitor. By 24 hpi over 75% of the cells undergoing caspase-independent cell death had permeable membranes. The results from both Hoechst and PI staining are consistent with a shift from PCD to necrosis when caspase inhibitors are present at the time of infection. While this shift might be predicted, the rapid cell death that ensued was not foreseen. As described above, MNV uses a novel mechanism for inducing PCD that involves down regulation of survivin. This suggests that PCD of the host cell is somehow advantageous for viral replication. Virus titers in cell media collected 24 hpi from cells infected in the presence or absence of polycaspase inhibitor revealed a reduction in titer from 7.5 × 10^6 ^to 5.1 × 10^5 ^pfu/mL, demonstrating that when PCD pathways were activated, virus replicated to higher yields.

### Cathespin B is activated by viral infection

**Figure 6:**
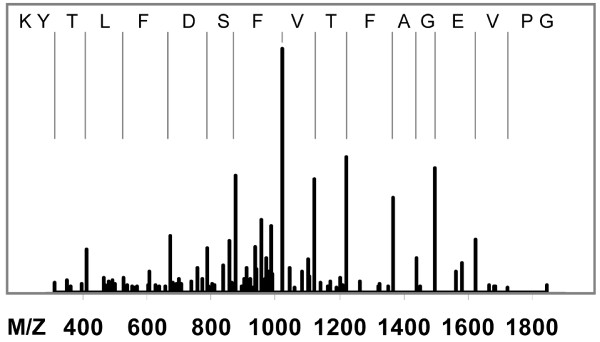
**Cathepsin is activated early by MNV-1 infection**. Fragmentation of tryptic peptides in an ion trap mass spectrometer was used to identify proteins labeled by a poly-caspase probe. This spectrum shows one of two tryptic peptides identified from Cathepsin. Amino acid sequence is shown in reverse order (C to N terminus) to match the Y ion series highlighted by the vertical bars. Ten B ions are also present but not marked. The match was highly significant in Mascot (p = 4.7 × 10^-10^) and Phenyx (p = 2.0 × 10^-57^) protein identification searches.

The chemical tags used in this study to inhibit/tag caspases and fluorometric caspase substrates are widely used to study apoptosis. Our use of specific and nonspecific probes raised the question of which protein was responsible for the strong polycaspase signal observed (Figure 3) because intrinsic apoptotic pathways involve caspase 9 and then 3, the latter of which came up at the later time point. The early activation after infection (Figure 1) was also of interest, because the time course of PCD is usually slower. These two points, along with the recent report that MNV uses a novel PCD pathway involving surviving [29] suggest that an unexpected enzyme could be responsible for our observed activity. To test this, biotin-X-DEVD-FMK was used so that labeled proteins could be retrieved using an affinity column. Infected cells were incubated with the activity probe and then the total cell lysate was passed over streptavidin-coated beads. The beads were washed three times with PBS followed by three high ionic strength washes (500 mM NaCl) to remove nonspecific binders. Streptavidin beads were then transferred to 50 mM Tris-HCl pH 8.0 and incubated overnight with trypsin. Peptides released from the bead bound proteins were directly analyzed using LC-MS/MS. Cathepsin B and a low level keratin signal were identified by MASCOT and Phenyx searches of the mouse and non redundant (nr) databases (Figure 6). Caspases were not identified in these experiments which could be a reflection of the number of proteins per cell, or the state of activation at the time point tested. Cathepsin B is a lysosomal protease and member of a family of 12 cysteine proteases [32,33]. Interestingly, cathepsin B has been shown to induce apoptosis both dependent and independent of caspase activation [32,34-36]. It can thus function as both an initiator and executioner protease that acts upstream of cytochrome C release from mitochondria. Lastly, this protein has been shown to play multiple roles during viral infection such as PCD signaling with human papilloma virus [37] and uncoating of adeno-associated viruses 2 and 8 [38] and is induced by grass carp hemorrhage [39], and influenza A viruses [20,24,40].

## Discussion

The connection between infection and apoptosis is well described for a number of viruses [19-27]. With respect to viruses in the *Caliciviridae*, feline calicivirus activates caspase 3 [27] and caspase 3 cleaves the MNV polyprotein during viral replication [25]. MNV infection also induces apoptosis, however, the process appears to be mechanistically distinct. Previously it was shown that MNV infection led to activation of the intrinsic PCD pathway with cytochrome C release, caspase 3 and 9 activation and DNA fragmentation [10]. The data presented in this study confirms these results using different methods and extends them to show that activation of caspase-like proteins occurs within two hpi, broad specificity caspase inhibitors accelerate cell death, and cathepsin B, an alternative protease in PCD identified by activity based screening was activated by infection.

Morphological changes observed in RAW264.7 cells upon MNV infection were consistent with those previously reported. Cell morphology, PI, and Hoechst staining showed that cells infected in the presence of a polycaspase inhibitor, Boc-D(OMe)-FMK, follow a distinctly different mechanism of cell death than cells infected in the absence of the inhibitor (Figure 5). In the absence of the caspase inhibitor, a high percentage of infected cells have condensed chromatin, and greater than 80 percent have intact membranes, which is consistent with the morphological changes associated with apoptosis. In the presence of the caspase inhibitor, infected cells show morphological signs of necrosis; very few cells have condensed and the majority has compromised plasma membrane integrity. These findings provide evidence that RAW 264.7 cells infected with MNV naturally undergo caspase-mediated apoptosis, and that caspase inhibitors alter the infection process leading to a cell death more closely resembling necrosis.

Caspase activation occurs downstream of the mitochondrial release of cytochrome c, and does not occur until 8 hpi during FCV infection [10]. Live cell imaging with a membrane permeable activity probe indicated activation occurred at a much earlier time point in MNV infected cells. Specifically, the polycaspase FLICA probe clearly showed positive labeling 2 hours after infection (Figure 1). This suggests that either caspases become activated much earlier during MNV infection of RAW264.7 cells, or that there is some off target labeling by the probe. The data presented in figure 3, shows that the polycaspase FLICA signal is greater than the sum of the specific probes. The extra signal in this channel could potentially be contributed to caspases that were not tested (1, 4, 6, 10, and 13), however, based on the previous FCV and MNV results this seems unlikely. We believe that the extra signal resulted from off-target labeling. This is strongly supported by the positive identification of cathepsin B by affinity purification of proteins labeled during infection. Other groups have suggested that caspase inhibitors are not highly specific and can target other proteases such as cathepsins [41-44]. While off-target labeling is a significant limitation for traditional uses of such molecules, it is an advantageous property when combined with affinity purification and peptide mass finger printing to identify other active proteases that may play a role in the infection. Regardless of whether the labeling is on or off-target, the enzyme must be catalytically active for covalent modification to occur. Figure 3 also shows that the more specific caspase probes are not subject to the same cross reactivity and activation of caspase 3; this follows closely with the recently reported data for MNV [29].

The response of murine RAW264.7 cells to MNV infection clearly involves PCD. It is also apparent that a noncanonical activation pathway for PCD using survivin and cathepsin B is involved. This program is initiated within hours of infection, leading to eventual cell death 24-36 hpi. In the presence of a pan-caspase inhibitor, cell death is accelerated and has the hallmarks of necrosis. A substantial reduction in the production of infectious virions occurs in this case - 1-1.5 logs. Numerous viruses take advantage of the biochemical events of PCD to process polyproteins, evade immune cell detection, and aid in egress. In the case of MNV, more virus is produced when PCD is allowed to progress. While FCV takes advantage of caspase 3 to process the polyprotein [25], it is not known if a similar mechanism is used by MNV. An explanation that is consistent with the available data is that MNV has adopted a strategy in which PCD is allowed to proceed, providing the virus a larger window of time for replication in comparison to the rapid necrosis that occurs in the absence of PCD. It is possible that these results are specific to MNV infection of RAW 264.7 cells, or could be the result of complex interactions between signaling pathways that are at odds if MNV infection and caspase inhibition are concurrent. This hypothesis can be tested and in the process should shed more light on the noncanonical PCD pathways activated by MNV and the biological significance of this. Furthermore, this work highlights the significant power of activity-based protein profiling in the study of viral pathogenesis.

## Materials and methods

### Cell Culture

RAW264.7 cells were cultured in Dulbecco's Modified Eagle's Medium (DMEM) supplemented with 10% premium-select FBS, 10 mM HEPES, 2 mM L-glutamine, and 25,000 units each of penicillin and streptomycin (complete DMEM). Cells were grown at 37°C with 5% CO_2_. Passage number for the cells was kept consistent throughout the experiments by returning to frozen ATCC stocks as needed.

### Qualitative Caspase Activity

Caspase activity was qualitatively monitored in infected and mock-infected cells during the first 12 hours of infection using a fluorescent SR-VAD-fmk poly caspase probe. Live cell labeling was done according to the FLICA manual (Immunochemistry Technology, Inc.) with slight modifications. RAW 264.7 Cells were seeded in 1.0 mL of cDMEM at a density of 1 × 10^6 ^cells per well in 12-well plates and allowed to grow at 37°C for one day then infected at an MOI of 3. Every two hours from 0 to 12 h.p.i. one infected and one mock-infected well were labeled with the FLICA, while one infected and one non-infected well were left unlabeled. The 0 (zero) time point was exposed to virus for 5 minutes prior to labeling. At each time point the media was aspirated and replaced with either 500 μL of cDMEM containing 1× FLICA (labeled) or 500 μL of cDMEM (unlabeled). Cells were incubated in the dark at 37°C for one hour; twice, the plates were gently tilted to distribute the probe. Cells were washed three times with 1.0 mL cDMEM for 5 minutes and 500 μL of fresh cDMEM were added to the cells before imaging. Cells were imaged directly in the plate wells using an inverted fluorescent microscope (Nikon TE300). Digital images of each sample at 400× magnification were taken and recorded using OneSpot software.

### Quantitative Caspase Activity

Caspase activity was quantitatively analyzed with a fluorometric assay using the FLICA-based probes (Immunochemistry Technologies, LLC) as described by the manufacturer. Activity levels for caspases 2, 3/7, 8, and 9 were quantified with FLICA probes in addition to the total caspase activity indicated from the pan-caspase FLICA probe. Probes included FAM-VDVAD-fmk (caspase-2), FAM-DEVD-fmk (caspase-3/7), FAM-LETD-fmk (caspase-8), FAM-LEHD-fmk (caspase-9) and SR-VAD-fmk (pan-caspase). A fluorometric plate reader was used to measure the relative fluorescent units (RFUs) per sample at 1, 5, 9, and 13 hours post infection.

At each time point (0, 4, 8, and 12 h) prior to labeling, cells were mock-infected or infected with MNV-1 at MOI = 3. Cells were harvested by scraping into 1.5 mL tubes 12 hours after the first inoculation. Cells were collected via centrifugation at 4,000 rpm for 10 minutes (2600 ×g). The supernatant was discarded and 300 μL of the appropriate 1× FLICA probe in DMEM was added to the cell pellet. Pellets were re-suspended by vortexing and incubated at 37°C for 1 hour with two brief vortexes to ensure adequate mixing for labeling. Samples were washed with 1.0 mL of a 1× wash buffer diluted in PBS and centrifuged at 4,000 rpm for 5 minutes (2600 ×g); the supernatant was aspirated and the wash was repeated. The pellets were re-suspended in 400 μL of PBS. Each sample was thoroughly mixed and 100 μL-aliquots were plated in triplicate in a 96-well plate. Fluorescence was measured with a fluorometric plate reader. Pan-caspase samples were measured with the 535/590 nm filter set, while all other samples were measured with the 490/535 nm filter set. Relative fluorescent units (RFUs) were recorded for each sample and normalized as a percentage of controls.

### Caspase-Independent Cell Death

Cell viability was monitored in infected cells in the presence and absence of the pan-caspase inhibitor and compared to their non-infected counterparts. Cells were seeded in 1.0 mL DMEM at a density of 5 × 10^5 ^cells per well in four 12-well plates and incubated at 37°C overnight. DMEM was replaced with 0.5 mL fresh medium plus 2.5 μL DMSO (control), DMEM with 100 μM Boc-D(OMe)-fmk (inhibitor control), DMEM with 2.5 μL DMSO and virus, DMEM with 100 μM Boc-D(OMe)-fmk and virus (inhibitor + virus). Each condition was repeated in triplicate for all four time points. At 0, 12, 18, and 24 h.p.i. non-contrast cell images were taken and percent viability was determined. Cells were imaged using an inverted Nikon TE300 microscope at 200× and 400× magnifications. Cells were then scraped, collected, and diluted 1:1 with Trypan Blue. 10 μL of the stained cells were counted with a hemacytometer to determine the total cell counts and percent viability.

### Cell Staining

Cells infected in the presence and absence of the pan-caspase inhibitor, Boc-D(OMe)-fmk, were stained with propidium iodide (PI) and Hoechst stain to measure apoptosis as described by Pelfry et al. Briefly, cells were seeded at a density of 5 × 10^5 ^cells per well in 1.0 mL DMEM in 12-well plates and incubated overnight at 37°C. Media was replaced with 0.5 mL DMEM plus virus (MOI of 3) for infected cells and 100 μM Boc-D(OMe)-fmk for caspase-inhibited cells. Infected were harvested at 6, 12, 18, and 24 h.p.i. for PI and Hoechst staining.

Cells were scraped, collected into 1.5 mL tubes, and centrifuged at 4000 rpm (2600 ×g) to pellet. The supernatant was aspirated and replaced with 250 μL of 1.6 μg/mL Hoechst stain diluted in sterile PBS. Cells were gently mixed with a 1000 μL-volume pipette tip and then incubated at 37°C for 15 minutes. 5 μL of 250 μg/mL PI was added to the cells and allowed to briefly incubate before centrifugation at 4000 rpm for 5 minutes (2600 ×g). The supernatant was discarded, and the cells were gently re-suspended in 20 μL of sterile PBS. 10 μL of cell suspension were plated onto a glass slide with a cover slip in preparation for imaging.

Cells were imaged on a Nikon TE300 inverted microscope. Digital cell images were taken at 400× magnification with each filter set and the images were merged using OneSpot software. Images were analyzed using ImageJ and a cell counter plug-in. Each image was individually analyzed for total cell counts and intensely stained cells. Hoechst-stained cells were counted and differentiated as having diffusely stained or intensely stained nuclei for each image. PI-stained cells were counted for the total number of membrane permeable cells per image. The percentage of cells with condensed chromatin was normalized to the control percentages for each time point to determine the virally induced changes in chromatin condensation.

### Affinity purification and protein identification

Cells were grown in 175 cm^2 ^flask until confluent and infected as previously mentioned. At 12 hpi, mock-infected and infected cells were harvested, pelleted, and re-suspended in 1.0 mL with 10 μM biotin-X-DEVD-FMK (Calbiochem Inc.) in cDMEM. One non-infected cell pellet was re-suspended in PBS alone to serve as a control. Cells were allowed to incubate at 37°C for one hour, pelleted, and washed once in 1× wash buffer. Cells were homogenized in 200 μL PBS binding buffer (0.15 M NaCl, and 0.1% SDS). 15 μL streptavidin agarose resin was then added to the cell lysate and mixed at room temperature for three hours. The resin was washed with 1.0 mL of binding buffer four times. Resin was washed once in PBS and digested with trypsin in PBS overnight at 37°C. Digested samples were analyzed with ESI-MS/MS, and protein identification was performed with MASCOT (Matrix Science) and Phenyx (GeneBio).

## Competing interests

The authors declare that they have no competing interests.

## Authors' contributions

LMF participated in the design and conducted the majority of the experiments in the study and helped to draft the manuscript. WSM and LP helped conduct the experiments and draft the manuscript. KE provided expertise for cell culture and handling of the virus and editing of the manuscript. MEH participated in the design and coordination of the study and helped to draft the manuscript. BB conceived of the study, participated in its design and coordination, and helped to draft the manuscript. All authors read and approved the final manuscript.
